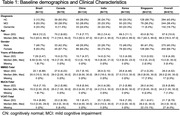# Visual Cognitive Assessment Test (VCAT): A language‐neutral test to detect mild cognitive impairment and dementia in multinational cohorts

**DOI:** 10.1002/alz.090328

**Published:** 2025-01-03

**Authors:** Kok Pin Ng, Gwen Cui Fann Ong, Seyed Ehsan Saffari, Marina Musse Bernardes, Antonella Brun de Carvalho, Lucas Porcello Schilling, Anandhi Ranganathan, Rema Raghu, Pedro Rosa‐Neto, Serge Gauthier, Yidan Liu, Xiaofeng Li, Min Jae Baek, SangYun Kim, Nagaendran Kandiah

**Affiliations:** ^1^ National Neuroscience Institute, Singapore, Singapore Singapore; ^2^ Duke‐NUS Medical School, Singapore Singapore; ^3^ National Neuroscience Institute, Singapore Singapore; ^4^ Brain Institute of Pontíficia Universidade Católica do Rio Grande do Sul, Porto Alegre Brazil; ^5^ Brain Institute of Rio Grande do Sul, PUCRS, Porto Alegre, RS Brazil; ^6^ Buddhi Clinic, Chennai, Tamil Nadu India; ^7^ Buddhi Clinic, Chennai India; ^8^ McGill University Research Centre for Studies in Aging, Montreal, QC Canada; ^9^ Translational Neuroimaging Laboratory, Montreal, QC Canada; ^10^ Department of Neurology and Neurosurgery, and Department of Psychiatry, McGill Centre for Studies in Aging, McGill University, Montreal, QC Canada; ^11^ the second affiliated hospital of Chongqing Medical University, Chongqing China; ^12^ The Second Affiliated Hospital of Chongqing Medical University, Chongqing, Chong Qing China; ^13^ Seoul National University Bundang Hospital, Seoul National University College of Medicine, Seongnam Korea, Republic of (South); ^14^ Lee Kong Chian School of Medicine, Nanyang Technological University, Singapore Singapore

## Abstract

**Background:**

Cognitive assessments are essential for the diagnosis of mild cognitive impairment (MCI) and dementia. However, existing tests are mostly developed in English‐speaking cohorts. Hence, their application in multilingual populations will need translation which may affect their test psychometrics. VCAT is a language‐neutral visual‐based assessment that is developed to address this issue. While VCAT was validated in Southeast Asian countries, its performance in diverse language cohorts remains unclear. Here, we aim to compare the utility of VCAT with established screening tests, Montreal Cognitive Assessment (MoCA) and Mini‐Mental State Examination (MMSE), in distinguishing MCI and dementia from cognitively normal (CN) individuals in a multinational study.

**Methods:**

This study supported by the Alzheimer’s Association has recruited 670 participants (294 CN, 244 MCI, 132 Dementia) from Brazil, Canada, China, India, Korea and Singapore and recruitment is ongoing. We standardized the administration of MMSE, MoCA and VCAT across all study sites. Participants answered a questionnaire on their demographics and underwent cognitive assessments (MMSE, MoCA and VCAT) on the same day. The performance of VCAT in distinguishing MCI and dementia from CN were assessed within each sites using the area under the curve (AUC) analysis.

**Results:**

The demographics, diagnosis and cognitive scores of the participants from each site were summarized in Table 1. The AUCs of VCAT in detecting MCI+Dementia vs CN were 0.979 for Brazil, 0.708 for Canada, 0.929 for China, 0.956 for Korea, 0.808 for India and 0.731 for Singapore. In comparison, the AUCs of MoCA in detecting MCI+Dementia vs CN were 0.771 for Brazil, 0.751 for Canada, 0.899 for China, 0.964 for Korea, 0.806 for India and 0.682 for Singapore, while the AUCs for MMSE in detecting MCI+Dementia vs CN were 0.896 for Brazil, 0.721 for Canada, 0.891 for China, 0.895 for Korea, 0.712 for India and 0.640 for Singapore.

**Conclusion:**

VCAT showed satisfactory discriminative validity in differentiating MCI+Dementia from CN participants within multinational, multilingual cohorts. VCAT was also comparable to the MoCA and MMSE. Further analysis in a larger cohort within our study will be performed to validate the utility of VCAT globally.